# Nicotinamide mononucleotide production by non-recombinant *Limosilactobacillus reuteri* and nicotinamide adenine dinucleotide synthesizing lactic acid bacteria

**DOI:** 10.1128/spectrum.00333-25

**Published:** 2025-10-20

**Authors:** Satoru Ozaki, Yoshiko Honme, Masashi Morifuji

**Affiliations:** 1 Wellness Science Labs, Meiji Holdings Co., Ltd., Tokyo, Japan; 2 Division of Research and Development, Meiji Co., Ltd., Tokyo, Japan; Institute of Microbiology, Chinese Academy of Sciences, Beijing, China

**Keywords:** nicotinamide mononucleotide, nicotinamide adenine dinucleotide, *Limosilactobacillus reuteri*, *Lactobacillus gasseri*, *Lactobacillus johnsonii*, *Lactobacillus delbrueckii*, NMN, NAD^+^

## Abstract

β-nicotinamide mononucleotide (NMN) is a precursor of nicotinamide adenine dinucleotide (NAD^+^) and has the potential to suppress aging. Certain strains of lactic acid bacteria (LAB) can produce cofactors and vitamins through fermentation. Dozens of LAB species are considered to be safe for human ingestion, and some strains act as probiotics, which are live microorganisms that confer health benefits to the host. However, the production of NMN by LAB species, especially non-recombinant strains, has rarely been investigated. The purpose of this study was to identify LAB species that produce NMN and to develop a method to obtain a higher concentration of NMN using LAB. We quantified NMN concentrations in the supernatants of MRS broth cultures of 18 LAB type strains and determined that *Limosilactobacillus reuteri* JCM 1112^T^ produced NMN. In this strain, the precursors of NMN were identified as NAD^+^, nicotinic acid, and nicotinamide based on the production of isotope-labeled NMN from these precursors labeled with stable isotopes. *L. reuteri* ME-989 produced NMN at 16.7 µmol/L by co-cultivation with *Lactobacillus johnsonii* JCM 2012^T^, which has a high NAD^+^-synthesizing ability. Non-recombinant *L. reuteri* and NAD^+^-synthesizing LAB may be useful for producing safe fermented products containing NMN.

**IMPORTANCE:**

β-nicotinamide mononucleotide (NMN) is a precursor of nicotinamide adenine dinucleotide (NAD^+^) and has the potential to suppress aging. Despite its increasing use in recent years, the production cost of NMN remains high. Certain strains of lactic acid bacteria (LAB), which are widely used in the fermentation of foods and are marketed as probiotics due to their safety and functional benefits, offer a promising alternative for the cost-effective production of functional compounds. However, the production of NMN by non-recombinant LAB has rarely been investigated except for some specific species. Our study has discovered that *Limosilactobacillus reuteri* produces NMN. The NMN production yield of this species was enhanced through co-cultivation with NAD^+^-synthesizing bacteria. These findings are significant for the development of safe, NMN-enriched fermented products.

## INTRODUCTION

Nicotinamide adenine dinucleotide (NAD^+^) is a cofactor of redox reactions in energy metabolism and a substrate for key enzymes of gene expression and DNA repair (1). In mice, *in vivo* NAD^+^ levels declined with aging (2, 3), and the supplementation of NAD^+^ precursors such as β-nicotinamide mononucleotide (NMN) improved energy metabolism, oxidative stress, and insulin sensitivity and mitigated physiological decline (4–6). In human clinical trials, NMN supplementation has been reported to improve insulin sensitivity, muscle function, quality of sleep, and walking speed (7–9). These reports have encouraged the use of NMN as a supplement, and industrial production methods for NMN have been developed.

Nucleotide compounds, such as NMN, have generally been produced by chemical or enzymatic methods, while microbial fermentative methods have been explored as cost-effective alternatives (10). Several pathways have been identified for NMN and NAD^+^ biosynthesis in bacteria, and the compounds directly converted to NMN are nicotinamide (NAM), nicotinic acid mononucleotide (NAMN), and NAD^+^ (11, 12). In NAD^+^ biosynthesis, nicotinic acid (NA), aspartic acid (Asp), and tryptophan (Trp) are utilized as precursors (13). Thus, NAM, NAMN, NAD^+^, NA, Asp, and Trp have the potential to act as precursors for NMN biosynthesis in bacteria.

Shoji et al. utilized recombinant *Escherichia coli* to biosynthesize NMN from NAM and phosphoribosyl pyrophosphate (PRPP) using the enzymatic activity of NAM phosphoribosyl transferase (NAMPT) (10). A high concentration (6.79 g/L ≈ 20.3 mmol/L) of NMN was obtained by the selection of highly active NAMPT and the enhancement of PRPP production in this report. The enzyme FtNadE, which is derived from *Francisella tularensis*, synthesizes NMN from NAMN (11, 14). *Lactococcus lactis* synthesized NAMN from NAM and NA, and the introduction of FtNadE into this organism enabled the production of NMN (11). These recombinant bacteria are effective in producing nucleotide cofactors. However, the intake of fermented products containing recombinant bacteria raises safety concerns, and removing these bacteria requires additional equipment, resulting in increased operational costs.

Lactic acid bacteria (LAB) are microorganisms that produce lactic acid from sugar. LAB have the potential to synthesize health-functional compounds, and some LAB strains can produce γ-aminobutyric acid, menaquinones, and antioxidative peptides (15–17). Dozens of LAB species have been consumed for a long time and are considered to be safe for human consumption by public agencies such as the European Food Safety Authority (18). Furthermore, some LAB strains function as probiotics, which improve the health of hosts (19–22).

Genus *Fructobacillus*, known as a fructophilic LAB, has been reported to produce NMN (23). This genus has NAMPT and produces NMN from NAM. However, the NMN yield from this organism was about 2 mg/L (≈6 µmol/L), which is less than the yield from recombinant bacteria. We have not identified any reports on NMN production by LAB, other than *Fructobacillus* and recombinant *L. lactis* (11, 23). Identifying LAB species that produce NMN may be useful for developing fermented products containing NMN.

The aim of this study was to identify LAB species that produce NMN. Eighteen type strains from 16 species were cultivated, and the NMN concentrations in the cultures were quantified. Furthermore, we investigated the NMN-producing pathway of LAB and developed an effective method to synthesize NMN.

## RESULTS

### NMN production by LAB species

**Fig 1:**
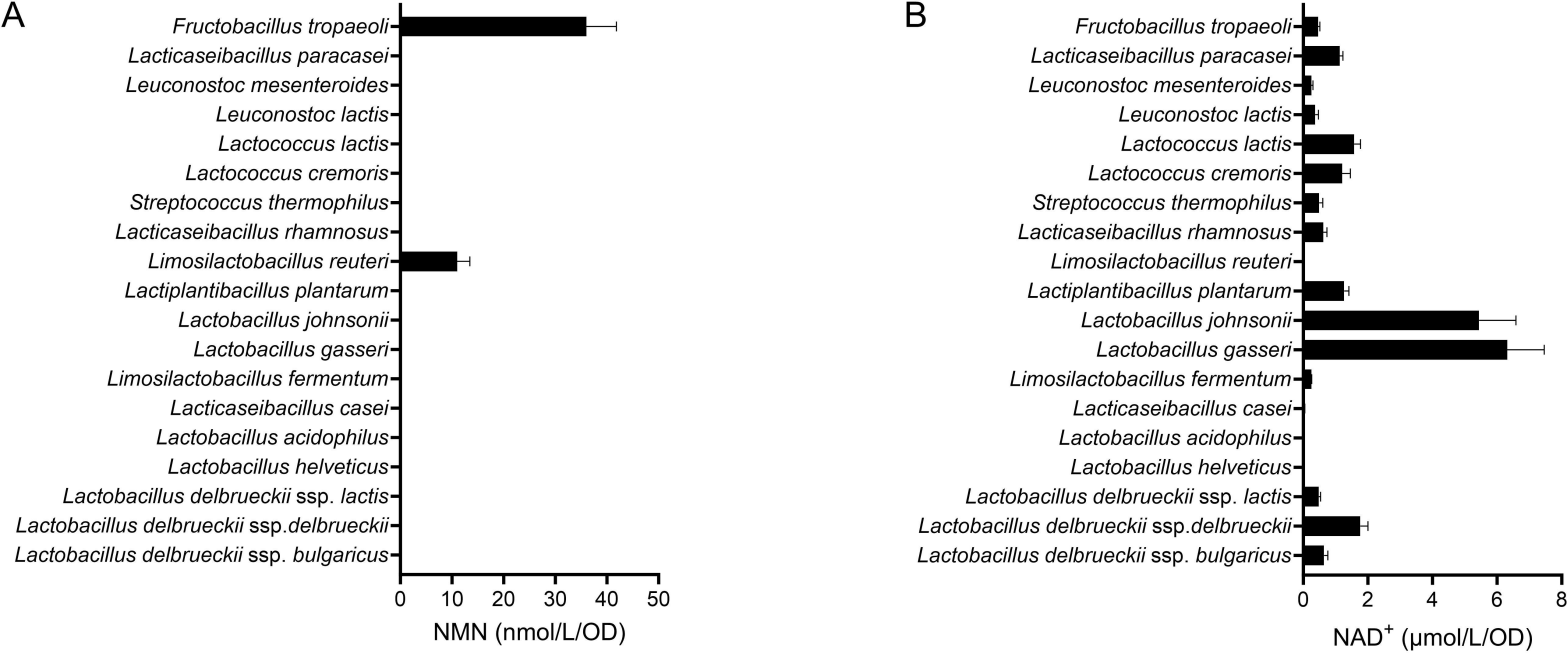
Concentrations of NMN and NAD^+^ in the supernatants of MRS broth cultures of LAB strains. NMN (**A**) and NAD^+^ (**B**) concentrations per OD_600_ unit are shown for each type strain and for *F. tropaeoli* ME-987. The cultivation time was 12 h. The black bars represent the mean values, and the error bars indicate the standard deviation calculated from triplicates.

To identify NMN-producing LAB species, we cultivated 18 type strains and ME-987, which belongs to the NMN-producing species *Fructobacillus tropaeoli* (23). The concentrations of NMN and related metabolites in the culture supernatants were quantified. *F. tropaeoli* ME-987 and *Limosilactobacillus reuteri* JCM 1112^T^ produced NMN at 36.0 and 11.0 nmol/L/OD_600_ unit, respectively (Fig. 1A). The NMN concentrations in the other supernatants and in the MRS broth were below the detection limit. NAD^+^ was detected in the supernatants of 15 strains but was not detected in MRS broth or in the supernatants of the other four strains, including *L. reuteri* (Fig. 1B). The concentrations of NAD^+^ in the supernatants of *Lactobacillus johnsonii* JCM 2012^T^ and *Lactobacillus gasseri* JCM 1131^T^ were 5.4 and 6.3 µmol/L/OD_600_ unit, respectively, which were much higher than those of the other strains. The concentration of NAM in the MRS broth was 24.9 µmol/L and those in the supernatants of the 13 strains, including *L. johnsonii* and *L. gasseri*, were below the detection limit (Table 1). The concentration of NA in the MRS broth was 18.6 µmol/L and increases in NA concentrations relative to the MRS broth were observed in the supernatants of 10 strains (Table 1). The NA concentrations in the supernatants of *L. johnsonii* and *L. gasseri* were below the detection limit.

**TABLE 1:** Concentrations of NAM and NA in the supernatants of MRS broth cultures of LAB strains^*a*^

	NAM		NA	
	µmol/L	Δµmol/L/OD	µmol/L	Δµmol/L/OD
*Fructobacillus tropaeoli*	23.3 ± 0.2	−0.6 ± 0.1	1.9 ± 0.1	−5.8 ± 0.1
*Lacticaseibacillus paracasei*	0.0 ± 0.0	−5.2 ± 0.3	18.6 ± 1.8	0.0 ± 0.4
*Leuconostoc mesenteroides*	26.4 ± 3.2	0.4 ± 0.8	0.0 ± 0.0	−5.0 ± 0.2
*Leuconostoc lactis*	34.8 ± 11.3	5.7 ± 6.4	8.0 ± 2.6	−6.2 ± 1.6
*Lactococcus lactis*	0.0 ± 0.0	−12.2 ± 0.3	30.1 ± 2.8	5.6 ± 1.3
*Lactococcus cremoris*	0.0 ± 0.0	−14.1 ± 0.2	37.3 ± 5.9	10.6 ± 3.4
*Streptococcus thermophilus*	13.6 ± 2.5	−5.8 ± 1.2	26.6 ± 5.7	4.1 ± 3.0
*Lacticaseibacillus rhamnosus*	0.0 ± 0.0	−2.9 ± 0.1	19.4 ± 4.9	0.1 ± 0.6
*Limosilactobacillus reuteri*	0.6 ± 0.0	−3.0 ± 0.1	19.1 ± 4.1	0.1 ± 0.5
*Lactiplantibacillus plantarum*	0.0 ± 0.0	−2.4 ± 0.1	3.3 ± 0.4	−1.4 ± 0.1
*Lactobacillus johnsonii*	0.0 ± 0.0	−3.8 ± 0.1	0.0 ± 0.0	−2.8 ± 0.1
*Lactobacillus gasseri*	0.0 ± 0.0	−3.8 ± 0.1	0.0 ± 0.0	−2.9 ± 0.1
*Limosilactobacillus fermentum*	0.0 ± 0.0	−3.7 ± 0.1	19.8 ± 2.8	0.2 ± 0.4
*Lacticaseibacillus casei*	37.1 ± 5.3	2.7 ± 1.2	0.0 ± 0.0	−4.1 ± 0.0
*Lactobacillus acidophilus*	0.0 ± 0.0	−3.6 ± 0.0	19.2 ± 1.5	0.1 ± 0.2
*Lactobacillus helveticus*	0.0 ± 0.0	−4.9 ± 0.2	28.3 ± 4.6	1.9 ± 0.9
*Lactobacillus delbrueckii* subsp. *lactis*	0.0 ± 0.0	−6.9 ± 0.2	30.2 ± 3.4	3.2 ± 1.0
*Lactobacillus delbrueckii* ssp. *delbrueckii*	0.0 ± 0.0	−6.2 ± 0.1	12.1 ± 0.3	−1.6 ± 0.1
*Lactobacillus delbrueckii* subsp. *bulgaricus*	0.0 ± 0.0	−7.2 ± 0.2	35.0 ± 6.0	4.8 ± 1.8
MRS broth	24.9 ± 6.4	–	18.6 ± 3.5	–

^*a*^ The table presents the concentrations of NAM and NA (µmol L⁻¹) together with OD_600_-normalized changes obtained by subtracting the background levels in uninoculated MRS broth (Δµmol/L/OD600 unit). The culture samples were identical to those described in Fig. 1. Each value represents the mean ± standard deviation of triplicate cultures. “–”, not applicable.

**Fig 2:**
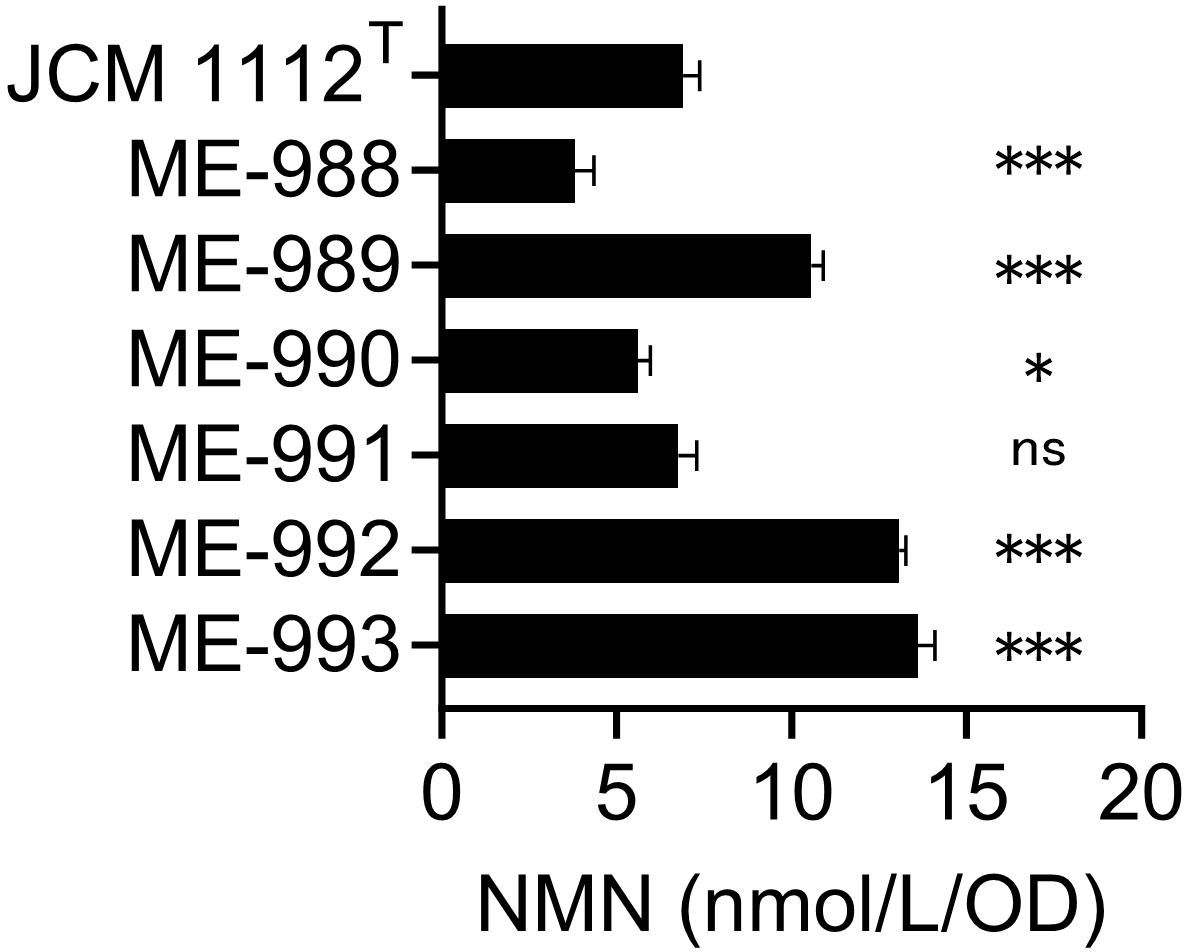
NMN concentrations in the supernatants of MRS broth cultures of *L. reuteri* strains. NMN concentrations per OD_600_ unit in the supernatants of MRS broth cultures of *L. reuteri* strains. The cultivation time was 12 h. The black bars represent the mean values, and the error bars indicate the standard deviation calculated from triplicates. One-way ANOVA showed a significant treatment effect (F(6,14) = 229, *P* < 0.0001). Post hoc Dunnett’s test was then performed using JCM 1112^T^ as the control; *P < 0.05, ***P < 0.001 versus control; ns = not significant.

To investigate NMN production by *L. reuteri* strains, the six strains isolated from human or mouse feces were cultivated in MRS broth (Fig. 2). The NMN concentrations in the supernatants of the six cultures ranged from 3.8 to 13.6 nmol/L/OD_600_ unit. The supernatants of ME-989, ME-992, and ME-993 showed significantly higher NMN concentrations than that of JCM 1112^T^. These results indicate that *L. reuteri* produces NMN and that this production is common in this species.

### The NMN-producing pathway of *L. reuteri*

**Fig 3:**
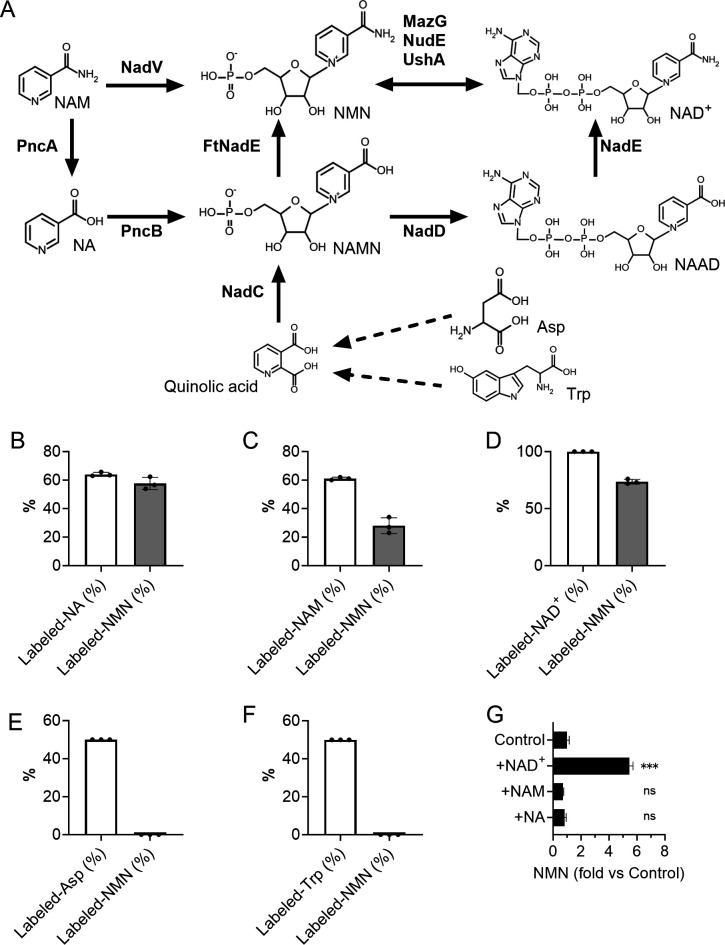
Production pathway and precursors of NMN in *L. reuteri* JCM 1112^T^. (**A**) Putative NMN production pathway in bacteria (11, 12). FtNadE: NMN synthase of *Francisella tularensis*; MazG: nucleoside triphosphate pyrophosphohydrolase; NadC: quinolinate phosphoribosyl transferase; NadD: NAMN adenylyl transferase; NadE: NAD⁺ synthetase; NadV: NAM phosphoribosyl transferase (NAMPT); NudE: NADH hydrolase; PncA: nicotinamidase; PncB: nicotinic acid phosphoribosyl transferase; UshA: UDP-sugar hydrolase. Dotted lines from Asp and Trp to quinolinic acid represent multiple reactions. (**B–F**) Production of labeled NMN by *L. reuteri* JCM 1112^T^ in MRS broth supplemented with ^13^C_6_-NA (**B**), ^13^C_6_-NAM (**C**), ^13^C_5_-NAD^+^ (**D**), ^13^C_4_-^15^N-Asp (**E**), or ^13^C_11_-^15^N_2_-Trp (**F**). The cultivation time was 12 h. The blank bars represent the percentages of labeled compounds (with respect to the total of labeled and unlabeled compounds) at the start of cultivation. The gray bars represent the percentages of labeled NMN (with respect to the total of labeled and unlabeled NMN) in the culture supernatants. The percentages of labeled NA, NAM, and NAD^+^ were calculated from the peak areas of each compound. The percentages of labeled Trp and Asp were calculated based on their respective concentrations of added labeled compounds and the unlabeled concentrations originally present in the MRS broth. (**G**) NMN concentrations in the supernatants of MRS broth cultures of *L. reuteri* JCM 1112^T^ supplemented with 5,000 ng/mL of NAD^+^ (7.5 µmol/L), NAM (41 µmol/L), or NA (41 µmol/L). The data are expressed as fold changes in NMN production relative to the unsupplemented MRS broth culture (Control). The cultivation time was 12 h. The bars represent the mean values, and the error bars indicate the standard deviation calculated from triplicates. One-way ANOVA showed a significant treatment effect (*F*(3,8) = 570, *P* < 0.0001). Dunnett’s test was then performed using unsupplemented MRS broth culture as the control; ***P < 0.001 versus control; ns = not significant.

NAM, NAMN, NAD^+^, NA, Asp, and Trp are potential precursors for NMN biosynthesis in bacteria (Fig. 3A) (11–13). To investigate the NMN production pathway in *L. reuteri* JCM 1112^T^, this strain was cultivated in MRS broth supplemented with isotope-labeled NAM, NA, NAD^+^, Asp, and Trp. Non-labeled and labeled NMN in the supernatants of the cultures were detected by liquid chromatography-tandem mass spectrometry (LC-MS/MS), and the percentage of labeled NMN was calculated. Labeled NMN was detected from the supernatants supplemented with labeled NA, NAM, or NAD^+^ (Fig. 3B through D), while it was not detected from those supplemented with labeled Asp or Trp (Fig. 3E and F). These results suggest that NA, NAM, and NAD^+^ are available to *L. reuteri* as precursors for NMN biosynthesis. To investigate which of the three compounds was the better NMN precursor for *L. reuteri*, JCM 1112^T^ was cultivated in MRS broth supplemented with NAD^+^, NA, or NAM, and NMN concentrations were quantified. To compare their precursor abilities, the resulting NMN levels were expressed as fold changes relative to the unsupplemented MRS culture (Fig. 3G). NMN concentration significantly increased in the medium supplemented with NAD^+^, but did not increase with NA or NAM compared to the original MRS medium. This result suggests that NAD^+^ is the best NMN precursor among the three compounds tested for *L. reuteri*.

**Fig 4:**
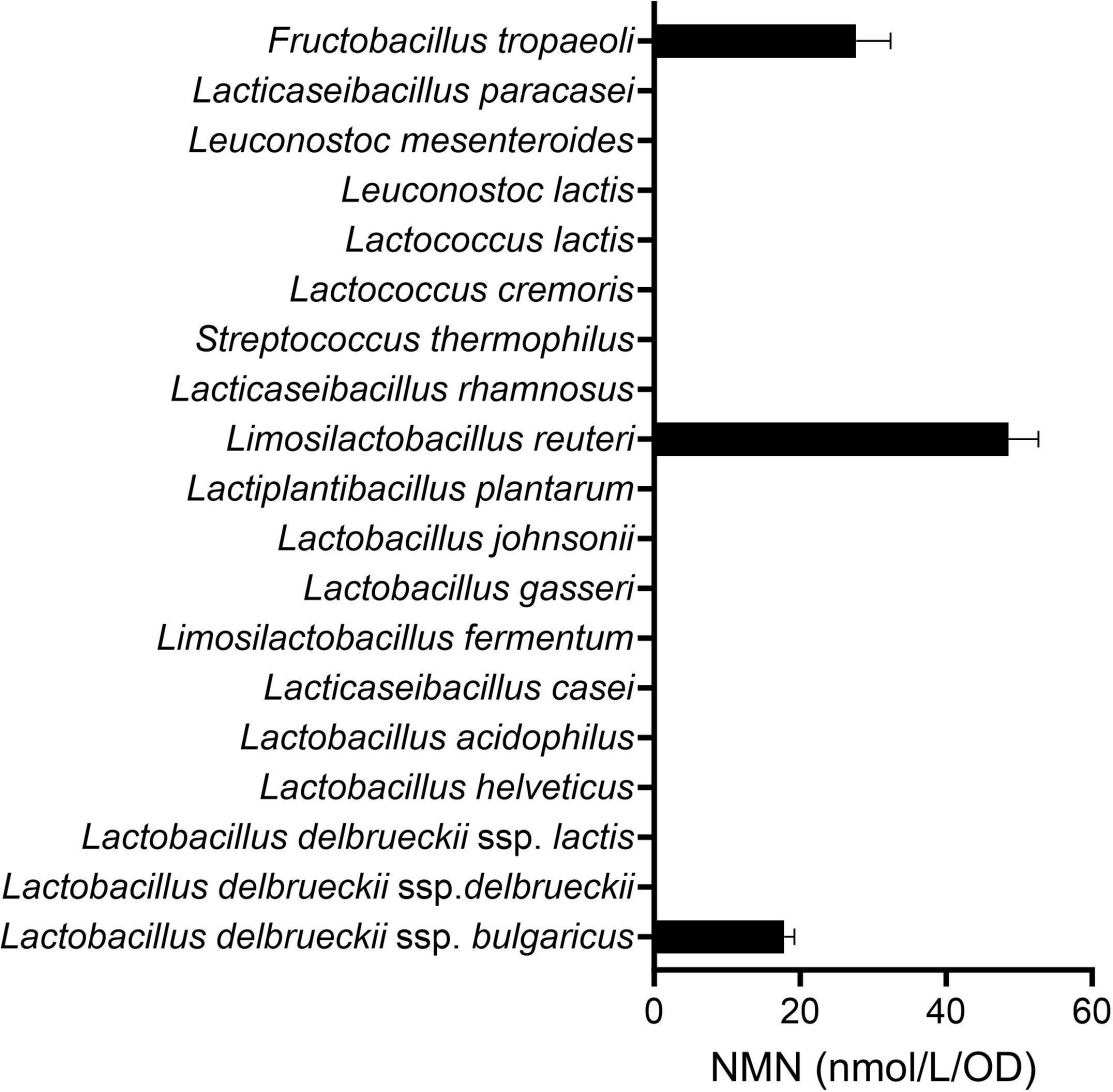
The effect of NAD^+^ supplementation on NMN production by LAB strains. NMN concentrations per OD_600_ unit in the supernatants of MRS broth cultures supplemented with 5,000 ng/mL (7.5 µmol/L) NAD^+^ are shown for each type strain and for *F. tropaeoli* ME-987. The cultivation time was 12 h. The black bars represent the mean values, and the error bars indicate the standard deviation calculated from triplicates.

To investigate the availability of NAD^+^ as an NMN precursor by other LAB strains, the LAB type strains and *F. tropaeoli* ME-987 were cultivated in the MRS broth supplemented with NAD^+^, and the NMN concentrations in the supernatants of the cultures were quantified. In addition to *L. reuteri* and *F. tropaeoli*, *Lactobacillus delbrueckii* subsp. *bulgaricus* JCM 1002^T^, which did not produce NMN in the MRS broth, synthesized NMN in the NAD^+^-supplemented broth (Fig. 4). These results suggest that the supplementation of NAD^+^ enabled *L. reuteri* JCM 1112^T^ and *L. delbrueckii* JCM 1002^T^ to efficiently biosynthesize NMN.

### Effect of co-cultivation with NAD^+^-synthesizing bacteria on the NMN production of *L. reuteri*

**Fig 5:**
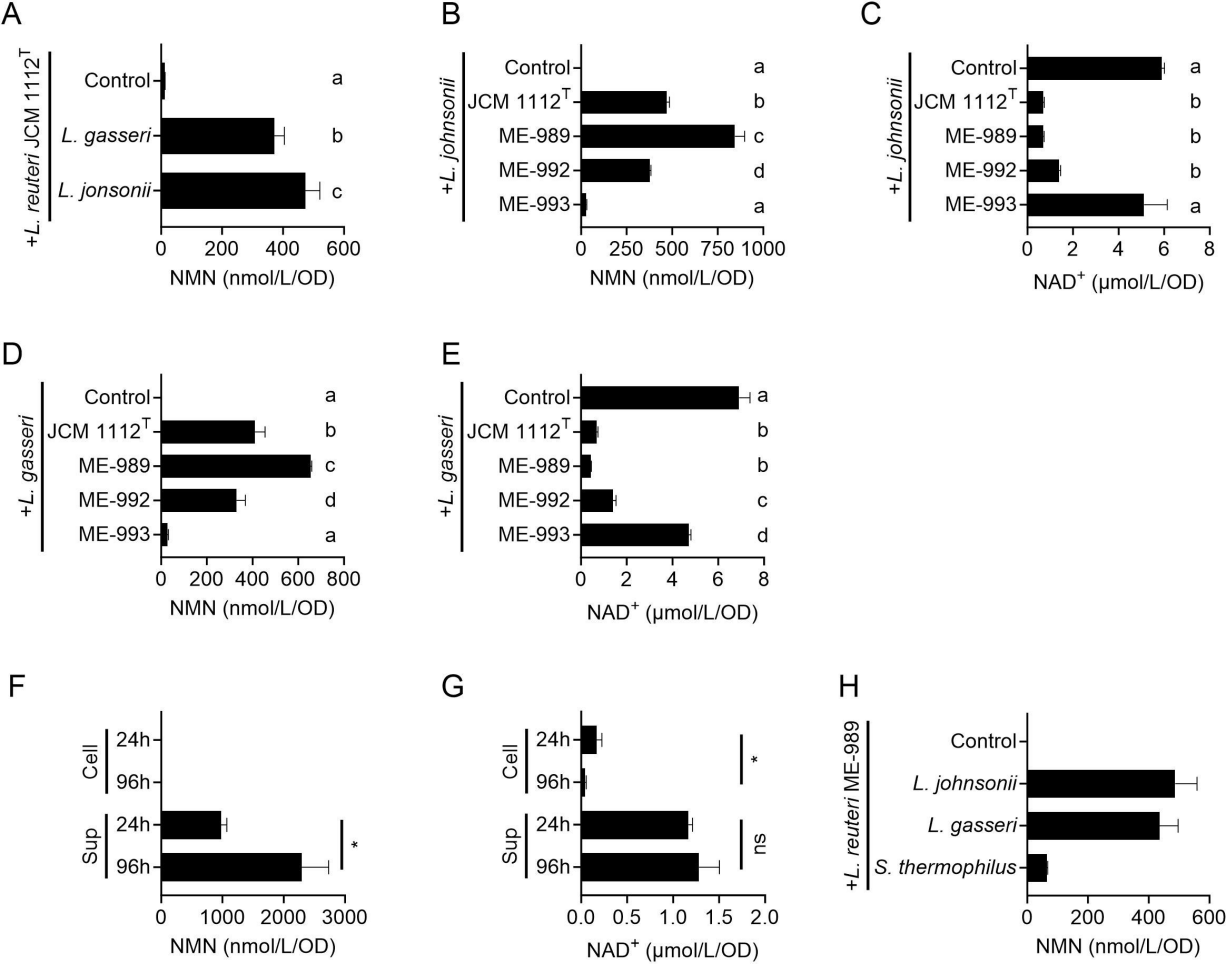
The effect of co-cultivation with NAD^+^-synthesizing bacteria on NMN production by *L. reuteri*. (**A**) Concentrations per OD_600_ unit of NMN in the supernatants of MRS broth cultures of *L. reuteri* JCM 1112^T^ with *L. gasseri* JCM 1131^T^ or *L. johnsonii* JCM 2012^T^. The control represents the concentration in the supernatant of the culture of *L. reuteri* JCM 1112^T^ only. The cultivation time was 12 h. One-way ANOVA indicated a significant treatment effect (*F*(2,6) = 154, *P* < 0.0001). Tukey’s multiple-comparison test was then performed; bars that do not share the same lowercase letter (**a–c**) differ significantly at *P* < 0.05, whereas bars with the same letter are not significantly different. (**B, C**) Concentrations per OD_600_ unit of NMN (**B**) and NAD^+^ (**C**) in the supernatants of MRS broth cultures of *L. reuteri* JCM 1112^T^, ME-989, ME-992, and ME-993 with *L. johnsonii* JCM 2012^T^. The control represents the concentration in the supernatant of the culture of *L. johnsonii* JCM 2012^T^ only. The cultivation time was 12 h. One-way ANOVA indicated significant treatment effects (*F*(4,10) = 176, *P* < 0.0001 [**B**] and *F*(4,10) = 91, *P* < 0.0001 [**C**]). Tukey’s multiple-comparison test was then performed; bars that do not share the same lowercase letter (**a–d**) differ significantly at *P* < 0.05, whereas bars with the same letter are not significantly different. (**D, E**) Concentrations per OD_600_ unit of NMN (**D**) and NAD^+^ (**E**) in the supernatants of MRS broth cultures of *L. reuteri* JCM 1112^T^, ME-989, ME-992, and ME-993 with *L. gasseri* JCM 1131^T^. The control represents the concentration in the supernatant of the culture of *L. gasseri* JCM 1131^T^ only. The cultivation time was 12 h. One-way ANOVA indicated significant treatment effects (F(4,10) =325, *P* < 0.0001 [**D**] and *F*(4,10) = 478, *P* < 0.0001 [**E**]). Tukey’s multiple-comparison test was then performed; bars that do not share the same lowercase letter (**a–d**) differ significantly at *P* < 0.05, whereas bars with the same letter are not significantly different. (**F, G**) Concentrations per OD_600_ unit of NMN (**F**) and NAD^+^ (**G**) in the supernatants (Sup) and the cell extracts (Cell) of MRS broth cultures of *L. reuteri* ME-989 with *L. johnsonii* JCM 2012^T^ grown with extended cultivation times of 24 and 96 h. Statistical differences between the 24 and 96 h time points were evaluated by Student’s *t*-test; *P < 0.05, ns = not significant. (**H**) NMN production through co-culture of *L. reuteri* and NAD^+^-producing bacteria in a medium composed of skimmed milk and yeast extract. *L. reuteri* ME-989 was cultivated with *L. johnsonii* JCM 2012^T^, *L. gasseri* JCM 1131^T^, and *S. thermophilus* ME-998. The cultivation time was 12 h. The black bars represent the mean values, and the error bars indicate the standard deviation calculated from triplicates.

Since NAD^+^ supplementation increased NMN production by *L. reuteri* JCM 1112^T^, this strain might produce NMN more efficiently when cultivated with NAD^+^-synthesizing strains. To investigate this possibility, JCM 1112^T^ was cultivated with either *L. johnsonii* JCM 2012^T^ or *L. gasseri* JCM 1131^T^, which produced high levels of NAD^+^ (Fig. 1B). The NMN production yields of JCM 1112^T^ with *L. johnsonii* and *L. gasseri* were 472 and 371 nmol/L/OD_600_ unit, respectively, which corresponded to 40- and 31-fold increases compared with the yield of JCM 1112^T^ alone (Fig. 5A). The yield with *L. johnsonii* was significantly higher than that with *L. gasseri*. Next, the effect of co-cultivation on NMN production was investigated with *L. reuteri* ME-989, ME-992, and ME-993, which all had higher NMN production than JCM 1112^T^ (Fig. 2A). In the case of the co-cultivation with *L. johnsonii*, the NMN concentration in the supernatant of the culture was the highest in ME-989, whereas the concentration of ME-992 and ME-993 was significantly lower than that of JCM 1112^T^, in contrast to monocultures (Fig. 5B). The concentration of NAD^+^ in the co-cultures of JCM 1112^T^, ME-989, and ME-992 was significantly lower than those in the monoculture of *L. johnsonii* and the co-culture of ME-993 (Fig. 5C), showing the opposite trend to that observed for NMN. Viable cell counts in the co-cultures were assessed, revealing that the co-culture of JCM 1112^T^ contained significantly more *L. reuteri* cells and fewer *L. johnsonii* cells than the co-culture of ME-993 (Table 2). In addition, the *L. reuteri* ratio in the co-cultures of JCM 1112^T^ and ME-989 was significantly higher than that in the co-cultures of ME-992 and ME-993. In the case of co-cultivation with *L. gasseri*, the highest NMN yield was observed for ME-989, and the trends of NMN and NAD^+^ concentrations in the supernatants of the four strains resembled those seen with *L. johnsonii* co-cultures (Fig. 5D and E).

**TABLE 2:** Viable cell counts in MRS broth cultures^*a*^

Strain	Individual culture	Co-culture with *L. johnsonii* JCM 2012^T^		
	(×10^9^ CFU/mL)	*L. reuteri* (× 10^9^ CFU/mL)	*L. johnsonii* (×10^9^ CFU/mL)	*L. reuteri* (%)
*L. reuteri* JCM 1112^T^	4.03 ± 0.75^a^	2.37 ± 0.84^a^	1.17 ± 0.38^a^	65.6 ± 16.4^a^
*L. reuteri* ME-989	3.10 ± 0.26^a^	1.36 ± 0.27^b^	1.27 ± 0.23^a^	51.8 ± 1.4^a^
*L. reuteri* ME-992	1.49 ± 0.16^b^	0.53 ± 0.01^b^	1.40 ± 0.19^ab^	27.7 ± 3.1^b^
*L. reuteri* ME-993	1.72 ± 0.12^b^	0.26 ± 0.04^b^	2.00 ± 0.14^b^	11.5 ± 1.6^b^
*L. johnsonii* JCM 2012^T^	2.38 ± 0.07	–	–	–

^*a*^ Each value represents the mean ± standard deviation of triplicate cultures. *L. reuteri* (%) indicates the percentage of *L. reuteri* cells relative to the total cell count in the co-culture. All one-way ANOVAs showed significant treatment effects (all *P* < 0.05). Superscript letters denote significant differences among means as determined by Tukey’s post hoc test (*P* < 0.05); values sharing the same letter are not significantly different. The cell count of *L. johnsonii *of the individual culture was excluded from the statistical analysis. “–”, not applicable.

The detection of NAD^+^ in all co-cultures (Fig. 5C and E) suggested that extending the cultivation time could increase the conversion of NAD^+^ to NMN. Thus, the effect of cultivation time on NMN production was investigated in the co-culture of ME-989 with *L. johnsonii*. The experiments described above employed a cultivation time of 12 h; the cultivation time was extended to 24 and 96 h. The NMN and NAD^+^ concentrations in the supernatants and cells were quantified. The NMN concentrations in the supernatants were 978 nmol/L/OD_600_ unit (8,900 nmol/L) and 2,293 nmol/L/OD_600_ unit (16,700 nmol/L) at 24 and 96 h, respectively (Fig. 5F). NMN was not detected in the cell extracts. NAD^+^ was detected in both the supernatants and the cell extracts (Fig. 5G). The concentration in the cell extracts was higher at 24 h than at 96 h, whereas that in the supernatants did not differ significantly between the two time points. In contrast, when the data were expressed as absolute concentrations (mol/L), NAD^+^ levels at 96 h were lower than those at 24 h in the supernatants (data not shown). These results indicate that co-cultivation with NAD^+^-synthesizing bacteria increased the NMN production of *L. reuteri*, and that the NMN production was enhanced by increasing the cultivation time. As an application example in fermented foods, we cultured *L. reuteri* ME-989 in a medium composed of skimmed milk and yeast extract, which are typical food production ingredients. ME-989 was cultured either alone or in combination with *L. johnsonii*, *L. gasseri*, or *S. thermophilus*, a species commonly used as a starter in fermented dairy products. *S. thermophilus* ME-998, which could produce NAD^+^ in this medium (data not shown), was used for the experiment. NMN production was observed under co-culture with all three strains (Fig. 5H). This result suggests that *L. reuteri* has the potential to produce NMN during the production process of fermented dairy products.

## DISCUSSION

We investigated NMN production by 18 type strains of 16 species of LAB and found that *L. reuteri* JCM 1112^T^ synthesized NMN (Fig. 1A). The NMN production by JCM 1112^T^ increased upon NAD^+^ supplementation or co-cultivation with NAD^+^-synthesizing bacteria (Fig. 3G and 5A). The maximum yield of NMN from *L. reuteri* was 16.7 µmol/L, achieved through the selection of the *L. reuteri* strain and the NAD^+^-synthesizing species and the use of a long cultivation time (Fig. 5F). This NMN yield was the highest among those of the non-recombinant LAB strains that we have investigated. These findings demonstrate the potential of *L. reuteri* as an NMN-producing bacterial species that does not require genetic modification. However, its NMN production was approximately 1,000-fold lower than that of recombinant *E. coli* (6.79 g/L, equivalent to 20.3 mmol/L) (10). To obtain a higher concentration of NMN, it will be necessary to understand the mechanisms of NMN production by *L. reuteri* and to develop strategies to enhance this production.

Several pathways have been identified by which bacteria produce NMN, and the compounds that are directly converted to NMN are NAM, NAMN, and NAD^+^ (Fig. 3A) (11, 12). Since JCM 1112^T^ produced isotope-labeled NMN in the broth that was supplemented with labeled NAD^+^ (Fig. 3D), we consider that this strain produces NMN from NAD^+^. The observations that supplementing NAD^+^ and co-cultivating with NAD^+^-synthesizing bacteria increased NMN production by *L. reuteri* also support the hypothesis that this species can produce NMN from NAD^+^.

Another NMN production pathway, namely the conversion of NAMN to NMN by the FtNadE enzyme, has been reported (14). However, we have not identified this enzyme activity in bacteria, with the exception of *F. tularensis*. The genus *Fructobacillus* has been reported to possess the enzyme NAMPT, which is encoded by *nadV* and synthesizes NMN from NAM (23). We could not identify the *nadV* gene in the *L. reuteri* genome by a search using *L. reuteri* and *nadV* as keywords in the database of the National Center for Biotechnology Information (NCBI Gene, https://www.ncbi.nlm.nih.gov/gene, accessed November 2024). Based on these investigations, we suspect that *L. reuteri* does not possess pathways that produce NMN directly from NAMN or NAM.

The pyrophosphatases encoded by the three genes *mazG*, *nudE*, and *ushA* are suspected to produce NMN from NAD^+^ (12). Based on the search of the NCBI Gene database, *L. reuteri* possesses only one of these three genes, *mazG*. This gene may be responsible for NMN production in *L. reuteri*. However, *L. johnsonii* and *L. gasseri*, which do not produce NMN, also have the *mazG* gene, based on analogous searches with NCBI Gene. To identify the reasons why only *L. reuteri* and *L. delbrueckii* subsp. *bulgaricus* exhibited NMN production, further research will be needed on the amino acid sequence, gene expression level, and enzyme activity of the pyrophosphatases encoded by *mazG* in each species.

*L. reuteri* also produced NMN from the precursors NAM and NA in addition to NAD^+^ (Fig. 3B and C). Since this species is not considered to have enzymes that produce NMN directly from NAM and NAMN, *L. reuteri* may produce NAD^+^ from NAM and NA, and then convert NAD^+^ to NMN. In MRS broth, the concentrations of NAM and NA were 24.9 and 18.6 µmol/L, and those in the culture of *L. reuteri* JCM 1112^T^ were 0.6 and 19.1 µmol/L, respectively (Table 1). The observation that NAM was almost exhausted and NA did not change appreciably during cultivation suggested that JCM 1112^T^ could convert almost all the NAM to NA in MRS broth within 12 h, while it took longer to produce NAD^+^ and NMN from NA. Also, supplementing NAD^+^ increased the NMN yield from JCM 1112^T^ (Fig. 3G), suggesting that the rate-limiting step of NMN production in this strain is the production of NAD^+^ from NA. On the other hand, *L. johnsonii* and *L. gasseri* produced over 5 µmol/L/OD_600_ unit of NAD^+^ and exhausted both NAM and NA within 12 h (Fig. 1B; Table 1). These results suggest that these two species can produce NAD^+^ from NAM and NA, and that the rate of NAD^+^ production of these two strains is much greater than that of *L. reuteri*. In co-cultivation, NAD^+^ synthesized by *L. johnsonii* or *L. gasseri* might increase the rate of NMN production of *L. reuteri*.

In individual cultures, *L. reuteri* ME-992 and ME-993 showed higher NMN concentrations than JCM 1112^T^ and ME-989 (Fig. 2A), but the opposite trend was observed in co-cultures (Fig. 5B and D). In co-cultures with *L. johnsonii*, the *L. reuteri* ratio of ME-992 and ME-993 was lower than that of JCM 1112^T^ and ME-989 (Table 2), suggesting that ME-992 and ME-993 might be weaker in competition for growth with other bacteria. These findings indicate that selecting strains strong in competition with other bacteria or combinations of strains with good compatibility may be important for increasing NMN production.

Optimizing NMN biosynthesis and excretion has been reported to be important for obtaining higher NMN yield in microorganisms (10–12). The observation that NAD^+^ was detected in the co-culture of *L. reuteri* ME-989 with *L. johnsonii* at 24 and 96 h suggested that the conversion of NAD^+^ to NMN was the rate-limiting step of NMN production. In the co-cultivation of four strains of *L. reuteri* with *L. johnsonii* or *L. gasseri*, as the concentration of NAD^+^ decreased in the co-culture, higher concentrations of NMN were obtained (Fig. 5B through E). These results suggest that the conversion rate of NAD^+^ to NMN is important to synthesize higher NMN concentrations in these cultures. PnuC excretes NMN from bacterial cells, and by introducing this gene into *E. coli*, the NMN yield improved (10). On the other hand, we could not detect NMN from cell extracts of the co-culture of *L. reuteri* with *L. johnsonii* (Fig. 5F). This suggests that NMN excretion is not the rate-limiting step of NMN production by *L. reuteri*.

In summary, we discovered that *L. reuteri* and *L. delbrueckii* subsp. *bulgaricus* can produce NMN. *L. reuteri* had a greater ability to convert NAD^+^ to NMN compared with other LAB species, and the NMN production by this species was improved by co-cultivation with NAD^+^-synthesizing bacteria.

The limitations of this study are that the number of strains was limited, and that we only examined static cultivation in MRS broth and food-grade medium. NMN production yield may be improved by the identification of strains that exhibit greater production of NMN and NAD^+^, and by the optimization of culture conditions. In addition, optimizing mixed-culture systems will require the development of strain-specific methods to enumerate viable cells for each organism. Further studies may be beneficial for producing fermented products containing NMN.

## MATERIALS AND METHODS

### Bacterial species and strains

Eighteen type strains of LAB were purchased from the Japanese Collection of Microorganisms (JCM). The strains were as follows: *Lactobacillus delbrueckii* subsp. *bulgaricus* JCM 1002^T^, *L. delbrueckii* subsp. *delbrueckii* JCM 1012^T^, *L. delbrueckii* subsp. *lactis* JCM 1248^T^, *Lactobacillus helveticus* JCM 1120^T^, *Lactobacillus acidophilus* JCM 1132^T^, *Lacticaseibacillus casei* JCM 1134^T^, *Limosilactobacillus fermentum* JCM 1137^T^, *L. gasseri* JCM 1131^T^, *L. johnsonii* JCM 2012^T^, *Lactiplantibacillus plantarum* JCM 1149^T^, *L. reuteri* JCM 1112^T^, *Lacticaseibacillus rhamnosus* JCM 1136^T^, *Streptococcus thermophilus* JCM 17834^T^, *Lactococcus cremoris* JCM 16167^T^, *L. lactis* JCM 5805^T^, *Leuconostoc lactis* JCM 6123^T^, *Leuconostoc mesenteroides* JCM 6124^T^, and *Lacticaseibacillus paracasei* JCM 8130^T^. *F. tropaeoli* ME-987 was isolated from kidney beans. *L. reuteri* ME-988, ME-989, and ME-990 were isolated from human feces. *L. reuteri* ME-991, ME-992, and ME-993 were isolated from mouse feces. *S. thermophilus* ME-998 was isolated from cow milk. Strains with numbers starting with “ME” were obtained from the culture collection of Meiji Co., Ltd.

### Cultivation of LAB and sampling

LAB strains were anaerobically cultivated in Lactobacilli MRS broth (Becton, Dickinson and Company, NJ, USA). For *F. tropaeoli* cultivation, fructose was added to the broth at a concentration of 2%. *Lactococcus* and *Leuconostoc* strains were grown at 30°C, while the other strains were grown at 37°C. An overnight culture was inoculated into 1 mL of the broth at a concentration of 1% (vol/vol) and incubated in a 96-well 2 mL deep well plate (Corning, NY, USA) unless otherwise specified. After incubation, the plate was centrifuged at 700 × *g* for 10 min at 4°C. The supernatant was dispensed onto a 0.22 µm hydrophilic filter plate (Millipore, France) and filtered by centrifugation at 700 × *g* for 10 min at 4°C. The filtrate was used for the quantification of compounds.

For the quantification of both intracellular and extracellular compounds, cultivation was conducted in 1.5 mL microtubes. After cultivation, the tubes were centrifuged at 20,630 × *g* for 10 min at 4°C and the supernatants were collected and filtered as described above. Cell extract preparation was carried out as described in the previous report (23) with slight modifications. Cells were washed twice with 0.85% potassium chloride and resuspended in 0.1 M potassium phosphate buffer (pH 7.2) containing a protein inhibitor cocktail (cOmplete EDTA-free, Roche Diagnostics, Germany). The suspension was disrupted with zirconia beads of 0.1 mm diameter (Toray, Japan) using FastPrep-24 (MP Biomedicals, CA, USA) and then centrifuged at 20,630 × *g* for 10 min at 4°C. The supernatant was filtered by the same method described above and used as a cell extract.

Skimmed milk (Meiji, Japan) at 20% (wt/wt) and yeast extract (Asahi Group Foods, Japan) at 0.1% (wt/wt) were dissolved in water and sterilized by autoclaving at 121°C for 7 min to prepare the food-grade medium. The inoculation and cultivation in this medium were conducted in the same manner as when using MRS broth.

All cultivation experiments were conducted independently in triplicate. Mean values and standard deviations were calculated for graphical representations.

### Cultivation in MRS broth supplemented with isotopically labeled compounds

For cultivation in MRS broth supplemented with isotopically labeled compounds, *L. reuteri* JCM 1112^T^ was grown in 1.5 mL microtubes. Either ^13^C_6_-NAM (Cambridge Isotope Laboratories [CIL], MA, USA), ^13^C_6_-NA (CIL), ^13^C_4_-^15^N-Asp (Sigma Aldrich, MO, USA), ^13^C_11_-^15^N_2_-Trp (Sigma), or ribose-^13^C_5_-NAD^+^ ammonium salt (CIL) was added to the broth at a concentration of 4.7, 2.3, 87, 207, and 5.0 µg/mL, respectively. The concentration of labeled NAM, NA, Asp, and Trp added to the broth was equivalent to the concentration of each unlabeled compound in MRS broth. The concentration of NAM and NA in the broth was quantified by LC-MS/MS as described below. The concentrations of Asp and Trp were quantified by the ω Scan package of Human Metabolome Technologies (Japan), using capillary electrophoresis Fourier transform mass spectrometry (24, 25).

### Metabolite quantification and normalization

Twenty microliters of filtered culture supernatants and cell extracts was mixed with 200 µL of cold 88.8% MeOH and set on ice for 30 min. When culturing in the food-grade medium, the culture was mixed directly with 88.8% methanol without centrifugation or filtration. The mixture was centrifuged at 20,630 × *g* at 4°C for 5 min, and 176 µL of supernatant was transferred to a new tube. The supernatant was evaporated in a centrifugal evaporator (miVac, ATS Genevac, UK) at 12 hPa at 35°C for 1 h. Evaporated samples were dissolved in 100 µL of a 10 mM ammonium acetate solution and then mixed with β-NMN-d_4_ (TLC Pharmaceutical Standards, Canada), ^13^C_6_-NAM, ^13^C_6_-NA, and ribose-^13^C_5_-NAD^+^ ammonium salt as internal standards for quantification using LC-MS/MS.

LC-MS/MS analysis was performed using a QTRAP 4500 mass spectrometer (AB Sciex, MA, USA) coupled with a Shimadzu HPLC system (Shimadzu, Japan). Chromatographic separation was achieved on a Hypercarb column (2.1 mm × 100 mm, 3 µm particle size, Thermo Fisher Scientific, WI, USA). The column temperature was maintained at 60°C. The mobile phase consisted of 7.5 mM ammonium acetate with 0.05% (vol/vol) ammonium hydroxide in water (A) and 0.05% (vol/vol) ammonium hydroxide in acetonitrile (B), and was delivered at a flow rate of 0.2 mL/min. The injection volume was 3 µL. The gradient elution started with 5% B for the first 1.8 min. This was followed by 54% B from 1.8 to 14 min, 90% B from 14.0 to 17.1 min, and a decrease to 5% B from 17.1 to 17.2 min. The concentration then remained at 5% B until 32.2 min. The MS/MS analysis was performed in the positive ionization mode. The detection of compounds was performed by multiple reaction monitoring. The ions were selected in the first quadrupole (Q1) and collided with nitrogen gas in the second quadrupole, and the product ions were detected in the third quadrupole (Q3). The *m/z* values of the precursor ions of NMN, NAM, NA, NAD^+^, NAMN, NMN-d4, ^13^C_6_-NAM, ^13^C_6_-NA, and ^13^C_5_-NAD^+^ detected at Q1 were 335.0, 123.0, 124.1, 664.2, 336.0, 339.1, 129.1, 130.1, and 669.1, and those of the product ions detected at Q3 were 123.0, 80.0, 78.1, 428.2, 123.9, 127.0, 85.0, 83.0, and 428.1, respectively. The detection of labeled NMN synthesized in MRS broth supplemented with ^13^C_6_-NAM, ^13^C_6_-NA, ^13^C_4_-^15^N-Asp, ^13^C_11_-^15^N_2_-Trp, or ^13^C_5_-NAD^+^ was performed with precursor ions of *m/z* values of 341.0, 341.0, 338.0, 342.0, and 340.0, and product ions of *m/z* values of 129.0, 129.0, 126.0, 130.0, or 123.1, respectively, predicted from the arrangement of isotopic elements in the labeled compounds. The detection limits of NMN, NAD^+^, NAM, NA, and NAMN were 180, 600, 2,400, 2,400, and 180 fmol, respectively. Normalization was performed by dividing the compound concentration (mol/L) by the OD at 600 nm, yielding values in units of mol/L/OD. The OD of the food-grade culture containing skimmed milk was measured according to the method of Morr (26), with the following modifications: 100 µL of culture or medium was mixed with 100 µL of 200 mM lactic acid and 800 µL of 10 M urea before measurement, to clarify the medium.

### Viable cell count of *L. reuteri* and *L. johnsonii*

The cultures were appropriately diluted with PBS and spread onto BL agar plates (Shimadzu Diagnostics, Japan). The plates were then incubated anaerobically at 37°C for 48 h. Among the appearing colonies, those with a circular shape were identified as *L. reuteri*, and those with a non-circular shape were counted as *L. johnsonii*.

### Statistical analysis

Statistical analyses were performed with GraphPad Prism v10 (GraphPad Software, CA, USA). For experiments involving more than two groups, a one-way analysis of variance (ANOVA) was conducted; post hoc comparisons were made with Dunnett’s test when comparing each group with the control, or with Tukey’s multiple comparison test when all pairwise contrasts were required. Differences between two independent groups were evaluated using an unpaired, two-tailed Student’s *t*-test. A *P* value < 0.05 was considered statistically significant.
